# HER2 targeting near‐infrared photoimmunotherapy for a CDDP‐resistant small‐cell lung cancer

**DOI:** 10.1002/cam4.4381

**Published:** 2021-11-02

**Authors:** Kazuomi Takahashi, Shunichi Taki, Hirotoshi Yasui, Yuko Nishinaga, Yoshitaka Isobe, Toshinori Matsui, Misae Shimizu, Chiaki Koike, Kazuhide Sato

**Affiliations:** ^1^ Respiratory Medicine Nagoya University Graduate School of Medicine Nagoya Japan; ^2^ B3 Unit Advanced Analytical and Diagnostic Imaging Center (AADIC)/Medical Engineering Unit (MEU) Nagoya University Institute for Advanced Research Nagoya Japan; ^3^ JST CREST FOREST‐Souhatsu Tokyo Japan; ^4^ S‐YLC Nagoya University Institute for Advanced Research Nagoya Japan

**Keywords:** chemotherapy‐resistant, cisplatin, HER2, near‐infrared photoimmunotherapy, small‐cell lung cancer

## Abstract

**Background:**

Human epidermal growth factor receptor 2 (HER2) is tyrosine kinase receptor that belongs to the ErbB family and is overexpressed on the membrane surface of various cancer cells, including small cell lung cancer (SCLC); however, no HER2 targeted therapy for SCLC have yet been established. Near‐infrared photoimmunotherapy (NIR‐PIT) is a novel cancer therapy based on photo‐absorber, IRDye‐700DX (IR700), ‐antibody conjugates, and near‐infrared (NIR) light.

**Methods:**

We used HER2‐positive SCLC parental cell lines (SBC‐3) and its chemoresistant cell lines, and examined therapeutic efficacy of HER2 targeting NIR‐PIT using anti HER2 antibody trastuzumab.

**Results:**

We found that HER2 expression was upregulated on chemoresistant cell lines, especially cisplatin‐resistance (SBC‐3/CDDP). In vitro, the rate of cell death increased with the amount of NIR‐light irradiation, and it was significantly higher in SBC‐3/CDDP than in SBC‐3. In vivo, tumor growth was more suppressed in SBC‐3/CDDP group than in SBC‐3 group, and survival period tended to be prolonged.

**Conclusion:**

In this study, we demonstrated that HER2 targeting NIR‐PIT using trastuzumab is promising therapy for HER2‐positive SCLC, and is more effective when HER2 expression is upregulated due to CDDP resistance, suggesting that the HER2 expression level positively corelated with the efficacy of NIR‐PIT.

## Graphical abstract

We found that human epidermal growth factor receptor 2 (HER2) was expressed in small‐cell lung cancer (SCLC) parental cell lines (SBC‐3) and its chemoresistant cell lines, and HER2 was upregulated in chemoresistant SCLC cells, especially CDDP‐resistant cells (SBC‐3/CDDP). The HER2‐targeting near‐infrared photoimmunotherapy (NIR‐PIT) using an anti‐HER2 antibody, trastuzumab, induced HER2‐expressing SCLC cells death, and efficacy of this therapy was significantly higher in SBC‐3/CDDP than in SBC‐3 in vitro and in vivo. These findings suggest a positive correlation between the cancer antigen expression level and the NIR‐PIT effect. Implications of all the available evidence. Our finding suggests that NIR‐PIT targeting HER2 using trastuzumab is a novel therapeutic approach for HER2‐positive SCLC when SCLC acquired CDDP‐based chemotherapy resistance and HER2 expression was upregulated.

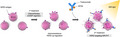

## 1 INTRODUCTION

Small‐cell lung cancer (SCLC) accounts for about 15% of lung cancers^1^ and is mostly found in the advanced stage due to early and widespread metastasis. However, there are only a few chemotherapy options for SCLC, and they have not changed significantly for 2–3 decades.^2^ The efficacy of combination therapy with cytotoxic drugs and immune checkpoint inhibitors has been reported, but it was not fully satisfactory.^3^, ^4^ The standard first‐line cisplatin (CDDP)‐based chemotherapy for SCLC has a good response rate. However, SCLC easily relapses within a few months in many cases due to acquired drug resistance to CDDP (refractory relapse). Although we have to change the treatment regimen other than CDDP‐based chemotherapy in that situation, few effective seconds or late line regimens are available. Therefore, a novel treatment for SCLC is needed after the failure of CDDP‐based regimens.^5^, ^6^, ^7^

Human epidermal growth factor receptor 2 (HER2) is a tyrosine kinase receptor that belongs to the ErbB family.^8^ HER2 forms homodimer or heterodimer with another ErbB family receptors on the cell membrane and regulates cancer cells differentiation, proliferation, and metastasis through signal transduction.^9^ HER2 is overexpressed in 13%–29.5% of SCLC and is a poor prognostic factor in extensive disease‐SCLC.^10^, ^11^ Several previous reports have suggested that HER2 expression is upregulated when SCLC is acquired chemoresistant.^12^, ^13^, ^14^ However, the clinical significance of HER2 overexpression in SCLC has not been fully clarified.

Trastuzumab, a humanized anti‐HER2 monoclonal antibody (mAb), has been used in the treatment of HER2‐positive breast cancer and gastric cancer.^15^, ^16^ Although several trials of trastuzumab for non‐small‐cell lung cancer (NSCLC) have been conducted, they have not shown significant efficacy. Several mechanisms of action of trastuzumab have been reported such as inhibition of HER2‐mediated signaling and antibody‐dependent cell‐mediated cytotoxicity.^17^ Recently, the novel HER2‐targeting antibody‐drug conjugates (ADC), trastuzumab deruxtecan, was approved and may have a positive impact on cancer treatment.^18^, ^19^ As above, the HER2‐targeting therapy has shown promise and will continue to be developed.

Near‐infrared photoimmunotherapy (NIR‐PIT) is the cancer photo‐targeted treatment based on photo‐absorber, IRDye‐700DX (IR700), antibody conjugates, and irradiation of near‐infrared (NIR) light.^20^ The photochemical reaction of NIR‐PIT rapidly induces necrosis, while it has little effect on nearby normal cells. Phase III randomized trials for locoregional recurrent head and neck squamous cell carcinoma with EGFR‐targeting NIR‐PIT using cetuximab‐IR700 (ASP‐1929) have been launched (NCT03769506), and ASP‐1929 was conditionally approved in Japan in 2020.

Herein, we evaluated the treatment effect of NIR‐PIT for HER2‐positive SCLC using trastuzumab and the correlation between the HER2 expression level and the efficacy of NIR‐PIT.

## 2 MATERIALS AND METHODS

### 2.1 Study design

The aim of this study is to develop a novel HER2‐targeting NIR‐PIT for HER2‐positive SCLC with trastuzumab, especially after the failure of CDDP‐based chemotherapy. All in vivo experiments were performed in compliance with the Guide for the Care and Use of Laboratory Animal Resources of Animal Care and Use Committee.

### 2.2 Reagents

IRDye 700DX NHS ester (IR700) was obtained from LI‐COR Bioscience. Trastuzumab (Herceptin^®^), a humanized IgG_1_ mAb for targeting HER2, was obtained from Chugai Pharmaceutical Co. Ltd.

### 2.3 Cell lines and cell culture

The human SCLC cell line SBC‐3 was purchased from the Japanese Collection of Research Bioresources (JCRB). The cell line is authenticated by the agency. The chemoresistant cell lines of SBC‐3, SBC‐3/CDDP, SBC‐3/ETP (etoposide), and SBC‐3/SN‐38 (7‐ethyl‐10‐hydroxycamptothecin) were kindly provided by Dr. Katsuyuki Kiura (Okayama University, Okayama, Japan).^21^, ^22^, ^23^ The luciferase‐expressing cell lines (SBC‐3‐luc and SBC‐3/CDDP‐luc) were established by transduction with RediFect Red‐FLuc‐Puromycin or RediFect Red‐FLuc‐GFP lentiviral particles (PerkinElmer), as described previously.^24^, ^25^ High luciferase expression was confirmed with more than 10 passages. RPMI‐1640 medium (Thermo Fisher Scientific) supplemented with 10% heat‐inactivated FBS, 100 IU/ml penicillin, and 100 μg/ml streptomycin (Thermo Fisher Scientific) at 37°C under 5% CO_2_ atmosphere were used for the cell line cultures.

### 2.4 Production of IR700‐conjugated trastuzumab

Trastuzumab (6.8 nmol) was mixed and incubated with IR700‐NHS ester (34.2 nmol) in Na_2_HPO_4_ (pH 8.5, 0.1 mol/L) for 1 h at 25°C. Sephadex G50 column (PD‐10; GE Healthcare) were used for the purification as described previously.^26^ Coomassie Plus protein assay kit (Thermo Fisher Scientific), with spectroscopically measuring the absorbance at 595 nm (Novaspec Plus; GE Healthcare), was used for the determination of the concentration of the protein. Measuring the absorbance at 689 nm to confirm the number of IR700 per antibody (dye‐mAb ratio) was done for the IR700 concentration. The synthesis was controlled so that the number of IR700 per antibody was approximately three.

### 2.5 SDS‐PAGE

SDS‐PAGE with 4%–20% Tris‐Glycine mini gel (Thermo Fisher Scientific) was used. After electrophoresis at 20 mA for 90 min, the fluorescence band on the gel was measured using a PEARL Imager (LI‐COR Bioscience) at 700 nm channels as described previously.^27^ Diluted trastuzumab was used as a control. The band on the gel with colloidal blue staining was measured to estimate the molecular weight of tra‐IR700.

### 2.6 Immunoblotting

About 5%–20% gradient gel was used for the SDS‐PAGE. The equal amounts of proteins were transferred to nitrocellulose membranes (Immobilon^®^‐FL; Merck Millipore) with Trans‐Blot Turbo (BioRad). The membranes were incubated with Rabbit anti‐HER2 polyclonal antibody (Protein Tech) as a primary antibody and anti‐actin antibody as an internal control (Wako) (diluted 1:500) overnight at 4°C, washed with TTBS, and incubated with secondary antibody (diluted 1:500) for 1 h at room temperature. The reactive bands on the gel were imaged using Odyssey Imager at 700 nm fluorescence channel.

### 2.7 Flow cytometry

The cells (1 × 10^5^) were incubated with tra‐IR700 (10 μg/ml) for 6 h at 37°C. The fluorescence intensity of IR700 was measured at 10,000 cell counts using a flow cytometer (Gallios, Beckman Coulter). A blocking study was performed to confirm that tra‐IR700 specifically binds to HER2. The SCLC cell lines were incubated with excessive amounts of unconjugated trastuzumab (100 μg) for 6 h at 37°C to saturate the binding ability of the HER2 receptor on the cell membrane, washed with PBS, and were incubated with tra‐IR700 (10 μg) for 2 h at 37°C.

### 2.8 Cell growth rate assay

The cells (1 × 10^5^) were cultured on 6‐well dishes and counted daily using a cell counter (TC20^™^; BioRad).

### 2.9 Fluorescence microscopy

The cells (1 × 10^4^) were cultured on 35‐mm glass‐bottomed dishes and incubated with tra‐IR700 (10 μg/ml) overnight at 37°C. After washing twice with PBS, the cells were irradiated with NIR light (4 J/cm^2^), stained with propidium iodide (PI) (final concentration was set to 2 μg/ml; Thermo Fisher Scientific) to detect the cell death,^28^ and observed using a fluorescence microscope (TiE‐A1R, Nikon Instech).^29^

### 2.10 In vitro NIR‐PIT

The cells (1 × 10^5^) were cultured on 12‐well dishes and incubated with tra‐IR700 (10 μg/ml) for 6 h at 37°C. After washing twice with PBS, the cells were irradiated with ranging 0–128 J/cm^2^ of NIR light‐emitting diode (L690‐66‐60; Ushio‐Epitex).^30^ An optical power meter (PM100; Thorlabs) was used for measuring the actual power density (mW/cm^2^) as described previously.^27^

### 2.11 In vitro cytotoxicity assay

In vitro, the efficacy of NIR‐PIT was determined via the luciferase activity and the rate of PI‐stained positive rate. For the luciferase assay, the luciferase‐expressing cells were treated with NIR‐PIT using tra‐IR700. After the incubation for 24 h, the luciferase‐expressing cells were washed with PBS, reacted with d‐luciferin media (150 μg/ml) (GoldBio), and analyzed with a plate scan reader (Powerscan 4; BioTek). For the PI staining assay, the cells were dissociated at 1 h after NIR‐PIT. The cell suspension was incubated with PI (final concentration was set to 2 μg/ml) for 30 min at 25°C. PI‐stained cells were measured by FACS Calibur (Becton Dickinson).

### 2.12 Xenograft tumor model

Eight to 10 weeks old female hemozygote athymic nude mice were commercially obtained from Chubu Kagaku Shizai. Isoflurane was used for the anesthesia during all procedures. The luciferase‐expressing cells (SBC‐3‐luc and SBC‐3/CDDP‐luc cells) (1 × 10^7^) were inoculated subcutaneously into the dorsum of mice. The estimated tumor volume was calculated as follows; tumor volume = length × width^2^ × 0.5. Tumors reached around 200 mm^3^ were randomized for the in vivo study. The mice were sacrificed using carbon dioxide asphyxiation when the tumor diameter reached 20 mm.

### 2.13 In vivo NIR‐PIT

Tra‐IR700 (100 μg) was administered intravenously to mice on day 1 (14 days after tumor cell transplantation on the dorsum). The tumors were irradiated with NIR light at 50 J/cm^2^ on day 0 and 100 J/cm^2^ on day 1. The antitumor efficacy of NIR‐PIT was determined with luciferase activity of the tumor, estimated tumor volume, and survival period.^31^

### 2.14 In vivo fluorescence imaging (FLI)

Tra‐IR700 was administered intravenously to mice via the tail vein and IR700‐fluorescence images were taken using a Pearl imager (LI‐COR Biosciences).^32^, ^33^, ^34^

### 2.15 In vivo bioluminescence imaging (BLI)

d‐luciferin media (7.5 mg/ml, 200 μl) was administered intraperitoneally to mice, and luminescence images were obtained 10 min later using IVIS imaging system (Perkin Elmer).^35^, ^36^ Luciferase activity of tumors was evaluated as the average radiance (p/s/cm^2^/sr) using analysis software (Living Image Software).

### 2.16 Statistics

Data are expressed as means ± SEM of a minimum of independent three experiments. Prism software (GraphPad) was used for statistical analyses. The unpaired *t*‐test was used for two‐group comparison, and the one‐way ANOVA with Friedman test with Dunn's multiple comparisons test was used for multiple‐group comparison. The Kaplan‐Meier analysis was performed to estimate the probability of survival defined as the nonachievement of tumor diameter of 20 mm, and the log‐rank and Wilcoxon tests were used to compare the results in each group. *p*‐values of <0.05 indicate a statistically significant difference.

## 3 RESULTS

### 3.1 Confirmation for tra‐IR700 conjugate

**FIGURE 1:**
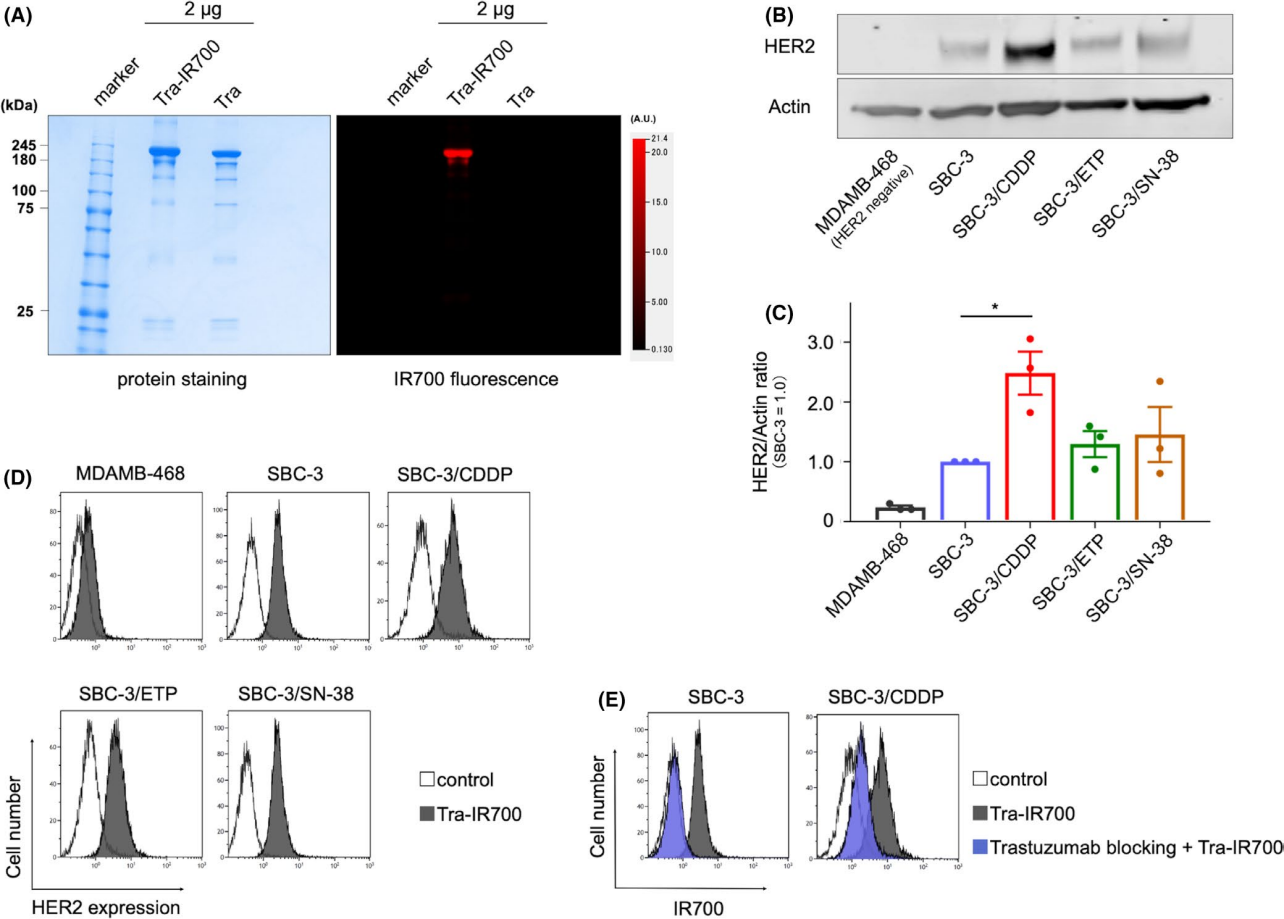
Synthesis of trastuzumab‐IR700 and evaluation of HER2 expression in SCLC cell lines. (A) Validation of trastuzumab‐IR700 (tra‐IR700) by SDS‐PAGE (left, Colloidal Blue stain: right, IR700‐fluorescence). Diluted trastuzumab (tra) was applied as a control. (B) HER2 expression in parental SCLC cells (SBC‐3) and chemoresistant SCLC cells (SBC‐3/CDDP, SBC‐3/ETP, and SBC‐3/SN‐38) were evaluated by western blotting. MDAMB‐468 was used as a negative control. (C) Western blot analysis of the ratio between HER2 protein and actin. HER2 expression tended to upregulate in SBC‐3/CDDP cells compared with that in SBC‐3 cells (*n* = 3, MDAMB‐468 vs. SBC‐3/CDDP; *p = 0.045 < 0.05, Friedman test with Dunn's multiple comparisons test) (see full immunoblot in Figure S1). (D) FACS analysis indicated specific binding of tra‐IR700 on HER2 in SBC‐3, SBC‐3/CDDP, SBC‐3/ETP, and SBC‐3/SN‐38 but not in MDAMB‐468. HER2 expression tended to upregulate in chemoresistant SCLC cells. (E) Specific binding of tra‐IR700 and HER2 was demonstrated with excess unconjugated trastuzumab in SBC‐3 and SBC‐3/CDDP. CDDP, cisplatin; HER2, human epidermal growth factor receptor 2; SCLC, small cell lung cancer

SDS‐PAGE was performed to confirm the conjugation between trastuzumab and IR700. The tra‐IR700 band appeared at slightly higher molecular weights than the nonconjugated control band (trastuzumab), and IR700‐fluorescence was observed only on tra‐IR700 (Figure 1A). These data indicated that tra‐IR700 was successfully produced.

### 3.2 HER2 expression on SCLC cells

HER2 expression on SCLC cells surface was evaluated by immunoblotting and flow cytometer to detect the binding tra‐IR700. HER2 is widely expressed in parental SCLC cells (SBC‐3) and chemoresistant SCLC cells (SBC‐3/CDDP, SBC‐3/ETP, and SBC‐3/SN‐38). Intriguingly, HER2 expression tended to upregulate in chemoresistant cells, which was evaluated with the immunoblotting (Figure 1B), especially in CDDP‐resistant cells (SBC‐3/CDDP) (Figure 1C and Figure S1) (*n* = 3, MDAMB‐468 vs. SBC‐3/CDDP; *p = 0.045 < 0.05, Friedman test with Dunn's multiple comparisons test). FACS analysis revealed tra‐IR700 specific binding to HER2 in SBC‐3, SBC‐3/CDDP, SBC‐3/ETP and SBC‐3/SN‐38 but not in MDAMB‐468. HER2 expression tended to upregulate in chemoresistant cells (Figure 1D). The excessive amounts of unconjugated trastuzumab inhibited tra‐IR700 binding to HER2 (trastuzumab blocking) in SBC‐3 and SBC‐3/CDDP (Figure 1E), suggesting that tra‐IR700 binds specifically to HER2. Based on these results, HER2‐targeting therapy for SCLC is considered more effective after the CDDP‐based chemotherapy, and we aimed to develop HER2‐targeting phototherapy.

### 3.3 Cell growth of SBC‐3 and SBC‐3/CDDP

**FIGURE 2:**
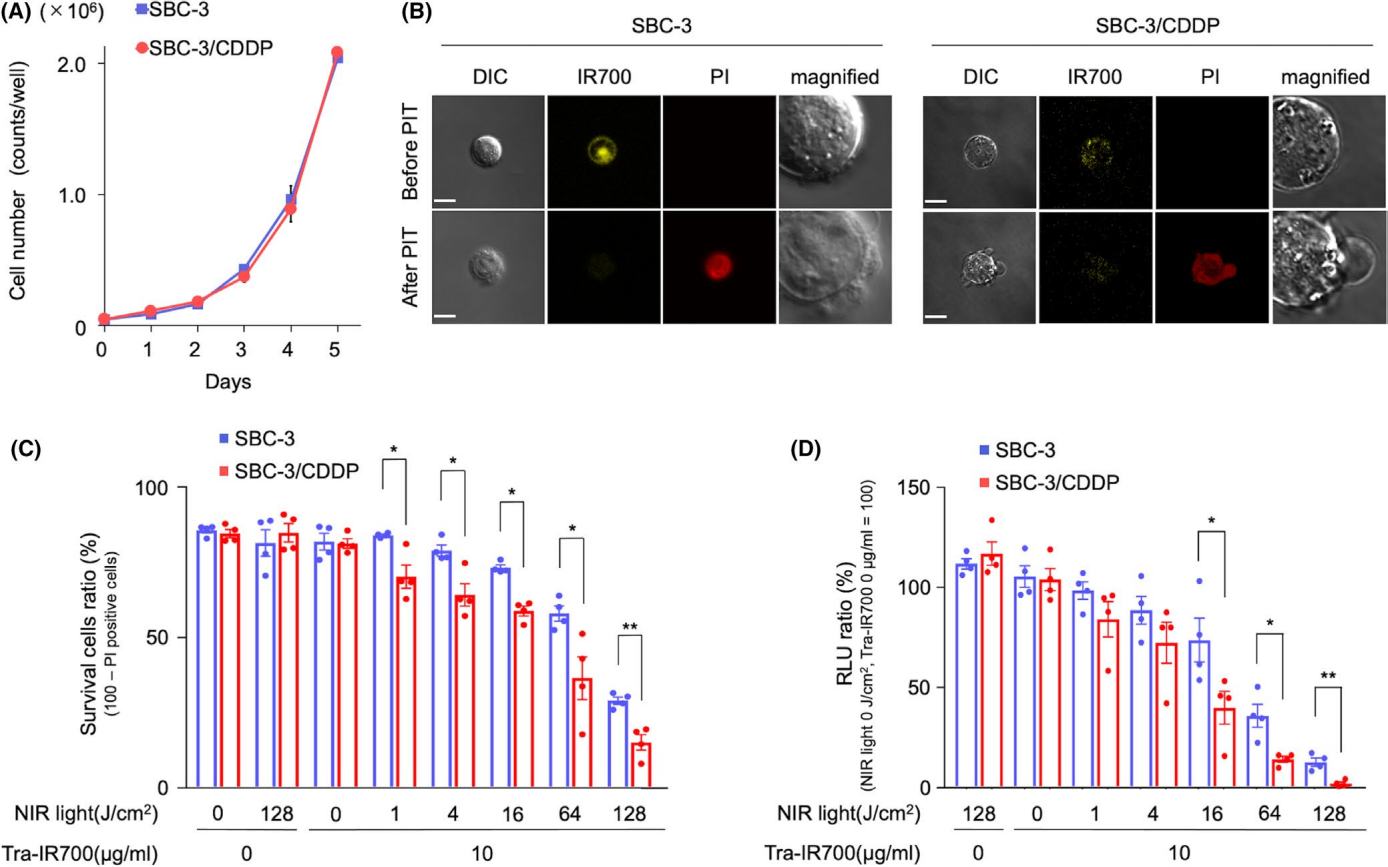
Comparative in vitro NIR‐PIT effectiveness between SBC‐3 and SBC‐3/CDDP. (A) SBC‐3 and SBC‐3/CDDP cells were counted daily using a cell counter. There was no difference in cell growth rate among the groups (data are shown as means ± SEM. *n* = 3, Student's *t*‐test). (B) SBC‐3 and SBC‐3/CDDP cells were treated with tra‐IR700 for 6 h and observed before and after NIR‐light irradiation (8 J/cm^2^) with a fluorescence microscope. Membrane damage and necrosis were detected after the Laser light (15 min after NIR‐PIT). Bar = 10 μm. (C) PI staining demonstrated cell death was increased in an NIR‐light dose‐dependent manner. There was a significant difference between the groups (data are means ± SEM. *n* = 4, *p < 0.05, **p < 0.001, Student's *t*‐test). (D) Luciferase activity measured as RLU showed that the cell viability was reduced in an NIR‐light dose‐dependent manner. A significant difference between the groups was detected (data are means ± SEM. *n* = 4, *p < 0.05, **p < 0.001, Student's *t*‐test). CDDP, cisplatin; NIR‐PIT, near‐infrared photoimmunotherapy; RLU, relative light units

The difference of the growth in SBC‐3 and SBC‐3/CDDP was assessed. The number of cells were measured daily with a cell counter, and cell growth was almost similar between the two (Figure 2A). These data indicated that CDDP resistance has no effect on the cell growth rate in SBC‐3/CDDP.

### 3.4 Microscopic observation of in vitro NIR‐PIT

Based on the prior results, we attempted to develop NIR‐PIT using the advantage of HER2‐upregulation on SBC‐3/CDDP. To measure the HER2‐targeting NIR‐PIT efficacy on SBC‐3 and SBC‐3/CDDP, we performed observation using fluorescence microscopy before and after NIR‐PIT. The IR700‐fluorescence of tra‐IR700 was detected on the cell surface and cytoplasm. After NIR‐PIT, the cells were swollen and finally burst. The fluorescence of PI, which meant necrotic cell, was observed, and simultaneously IR700‐fluorescence was attenuated after NIR‐PIT (Figure 2B). These changes were detected within 30 min after NIR‐light irradiation, suggesting that NIR‐PIT indicated rapid induction of necrotic cell death on both SBC‐3 and SBC‐3/CDDP.

### 3.5 In vitro NIR‐PIT effect with tra‐IR700 on SCLC cell lines

Next, the cytotoxicity assay based on both flow cytometric PI staining and measuring luciferase activity were performed using SBC‐3‐luc and SBC‐3/CDDP‐luc to compare the NIR‐PIT efficacy between SBC‐3 and SBC‐3/CDDP. The necrotic cells (PI‐positive cells) was increased in NIR‐light dose‐dependent, and was significantly higher in SBC‐3/CDDP‐luc than in SBC‐3‐luc (Figure 2C) (data are means ± SEMs [*n* = 4, *p < 0.05, **p < 0.001, Student's *t*‐test], *p* = 0.0119 [tra‐IR700 added + NIR light at 1 J/cm^2^], *p* = 0.0120 [tra‐IR700 added + NIR light 4 J/cm^2^], *p* = 0.0003 [tra‐IR700 added + NIR light 16 J/cm^2^], *p* = 0.0298 [tra‐IR700 added + NIR light 64 J/cm^2^], *p* = 0.0031 [tra‐IR700 added + NIR light 128 J/cm^2^]).

The luciferase activity of cells decreased in an NIR‐light dose‐dependent manner. The relative light units (RLU) of luciferase activity were significantly lower in SBC‐3/CDDP‐luc than in SBC‐3‐luc (Figure 2D) (data are means ± SEMs [*n* = 4, *p < 0.05, **p < 0.001, Student's *t*‐test], *p* = 0.0479 [tra‐IR700 added + NIR light 16 J/cm^2^], *p* = 0.0103 [tra‐IR700 added + NIR light 64 J/cm^2^], *p* = 0.0028 [tra‐IR700 added + NIR light 128 J/cm^2^]). These results suggested that HER2‐targeting NIR‐PIT using tra‐IR700 caused the cell death in HER2‐positive SCLC, and it was more effective in higher HER2 expression cells at the same light energy level. These results promoted us to investigate HER2‐targeted NIR‐PIT effect using in vivo SCLC tumor‐bearing mouse.

### 3.6 In vivo biodistribution of tra‐IR700

**FIGURE 3:**
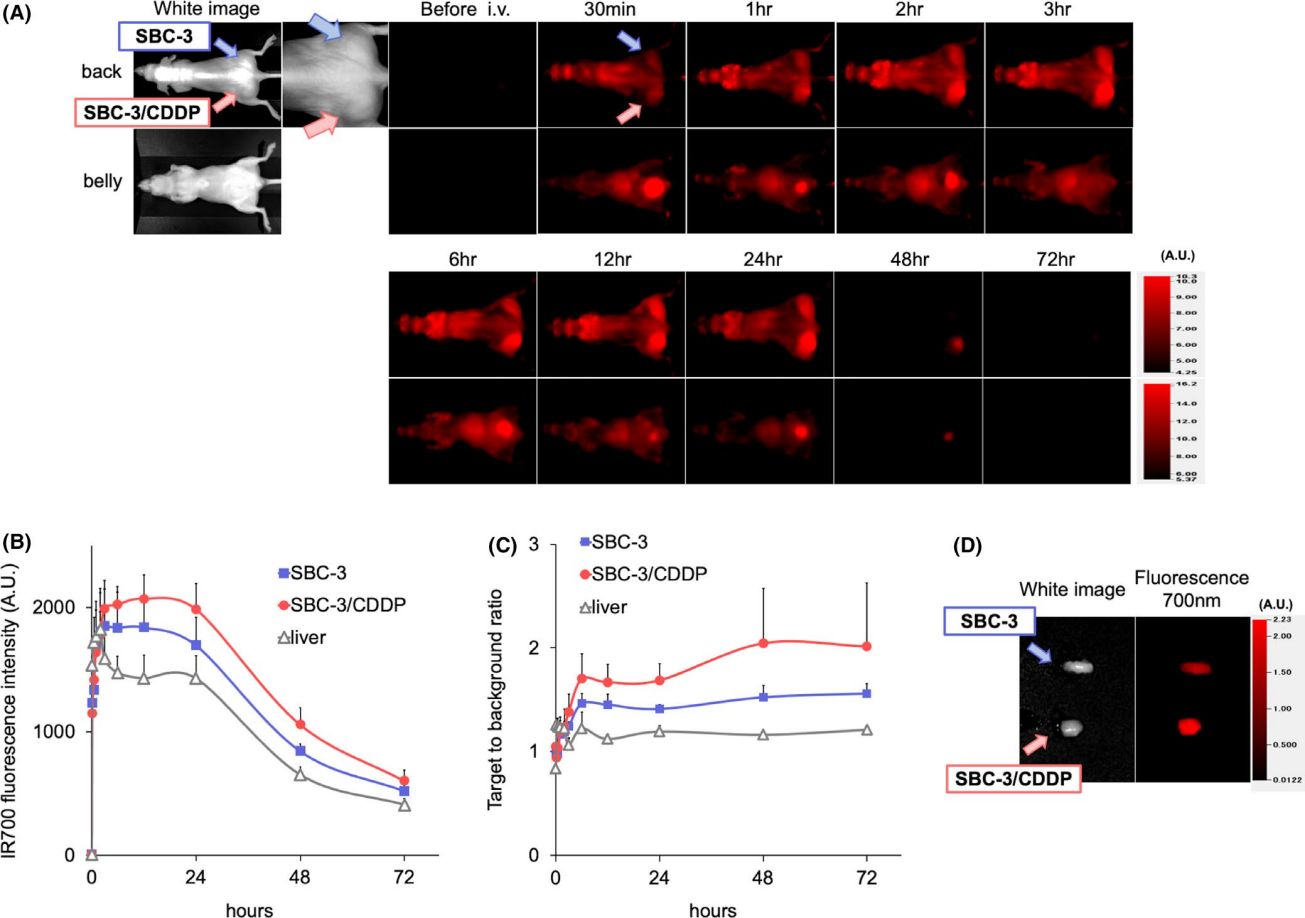
Comparing biodistribution of tra‐IR700 in SBC‐3 and SBC‐3/CDDP tumor‐bearing mice. (A) In vivo 700 nm fluorescence‐imaging (FLI) of the tumor‐bearing mice (right‐side dorsum: SBC‐3 tumor, left‐side dorsum: SBC‐3/CDDP tumor) using tra‐IR700. Both tumors showed high fluorescence signals after intravenous administration of tra‐IR700. The IR700‐fluorescence was higher in SBC‐3/CDDP tumors than in SBC‐3 tumors. (B) Quantification of 700 nm fluorescence intensities in the tumors and liver. Fluorescence intensities tended to be higher in SBC‐3/CDDP tumors than in SBC‐3 tumors at all time points. (data are means ± SEM. *n* = 3). (C) Target to background ratio (TBR) also tended to be higher in SBC‐3/CDDP tumors than in SBC‐3 tumors (data are means ± SEM. *n* = 3). (D) Ex vivo white light and FLI of SBC‐3 and SBC‐3/CDDP tumor at 24 h after injection of tra‐IR700. FLI showed higher IR700‐intensity in SBC‐3/CDDP tumors than in SBC‐3 tumors. CDDP, cisplatin

A subcutaneous xenograft tumor model (SBC‐3 tumor on the right side of the dorsum and SBC‐3/CDDP tumor on the left side of the dorsum) was established to evaluate in vivo biodistribution. After the intravenous injection of tra‐IR700 via a tail vein, an IR700‐fluorescence image (FLI) was acquired. Tra‐IR700 accumulated at tumors 30 min after the administration and almost peaked at 24 h (Figure 3A). Quantification of IR700‐fluorescence showed that the highest accumulation of tra‐IR700 on tumors was detected on day 1, whereas the highest tumor‐to‐background ratio (TBR) was observed on days 2 and 3 (Figure 3B,C). SBC‐3/CDDP tumor showed higher IR700‐fluorescence intensity than SBC‐3 tumor at each time point. No other specific accumulation of IR700‐fluorescence except for the bladder and liver, which was the process of metabolism and excretion, was detected. The accumulation of tra‐IR700 on tumors was confirmed with ex vivo imaging (Figure 3D). With these results, tra‐IR700 was specifically distributed on HER2‐expressing tumors, and that it was reasonable to perform NIR‐PIT on days 1 and 2 after administration of tra‐IR700. Moreover, the accumulation of intravenously injected tra‐IR700 was enhanced in HER2‐upregulated SBC‐3/CDDP tumor.

### 3.7 In vivo NIR‐PIT antitumor effect with tra‐IR700

**FIGURE 4:**
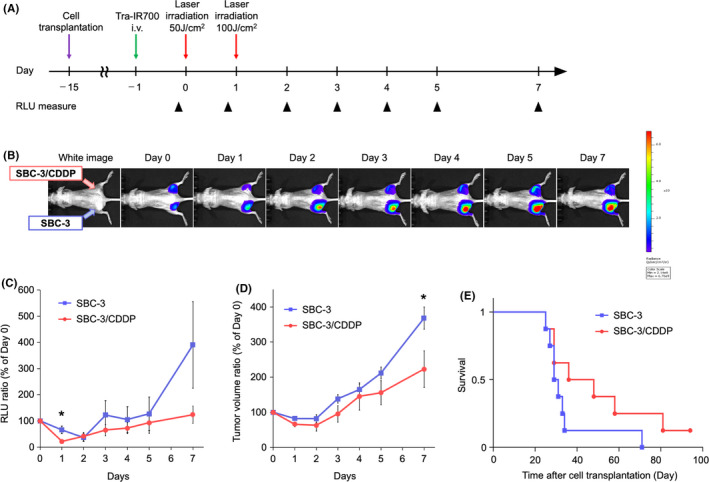
Comparing in vivo NIR‐PIT anti‐tumor effect in SBC‐3 and SBC‐3/CDDP tumors. (A) The treatment regimen and imaging schedule. Bioluminescence images (BLI) were taken as indicated. (B) BLI of SBC‐3 (left‐side dorsum) and SBC‐3/CDDP (right‐side dorsum) tumor‐bearing mice along with the treatment. Both groups were about the same size and exhibited similar bioluminescence intensity before NIR‐PIT treatment. (C) Quantitative luciferase activity (RLU value at day 0 is set to 100%) showed a decrease in both groups after NIR‐PIT treatment. RLU ratio was lower in SBC‐3/CDDP tumors than in SBC‐3 tumors (data are means ± SEM. *n* = 8, *p = 0.022 < 0.05, at day 1, Student's *t*‐test). (D) Tumor growth (estimated tumor volume at day 0 is set to 100%) was significantly inhibited in SBC‐3/CDDP tumors compared with SBC‐3 tumors (data are means ± SEM. *n* = 8, *p = 0.043 < 0.05, at day 7, Student's *t*‐test). (E) Kaplan–Meier analysis. SBC‐3/CDDP tumor group tended to have longer survival compared with the SBC‐3 tumor group, but no statistically significant difference was detected (*n* = 8, *p* = 0.124, log‐rank test). CDDP, cisplatin; NIR‐PIT, near‐infrared photoimmunotherapy; RLU, relative light units

Finally, the in vivo HER2‐targeting NIR‐PIT antitumor effect on SBC‐3 and SBC‐3/CDDP tumor were examined. The NIR‐PIT regimen was demonstrated in Figure 4A. The luciferase activity of tumors from in vivo BLI was measured in a subcutaneous xenograft tumor model (SBC‐3‐luc tumor on the left side of the dorsum and SBC‐3/CDDP‐luc tumor on the right side of the dorsum) (Figure 4B). The RLU ratio in BLI (day 0 was set to 100%) showed a decrease in both SBC‐3 and SBC‐3/CDDP tumors after NIR‐PIT, and the NIR‐PIT efficacy was significantly higher in the SBC‐3/CDDP‐luc tumor group than in the SBC‐3‐luc tumor group (Figure 4C) (data are means ± SEMs. *n* = 8, *p = 0.0221 < 0.05, at day 1, Student's *t*‐test). We also evaluated tumor volume in these mice. Both SBC‐3‐luc and SBC‐3/CDDP‐luc tumor volume was reduced after NIR‐PIT. SBC‐3/CDDP‐luc tumor volume were smaller than SBC‐3‐luc at each time point, significantly 7 days after (Figure 4D) (data are means ± SEM. *n* = 8, *p = 0.0430 < 0.05, at day 7, Student's *t*‐test). Survival period were prolonged in SBC‐3/CDDP group (Figure 4E) (each group *n* = 8, *p* = 0.1241, log‐rank test). These results suggested that HER2‐targeting NIR‐PIT have antitumor effects in HER2‐positive in vivo SCLC tumor models, and it is more effective in higher HER2 expression tumors at the same light energy level. Collectively, HER2‐targeting NIR‐PIT was rational on HER2‐upregulated CDDP‐resistant SCLC.

## 4 DISCUSSION

**FIGURE 5:**
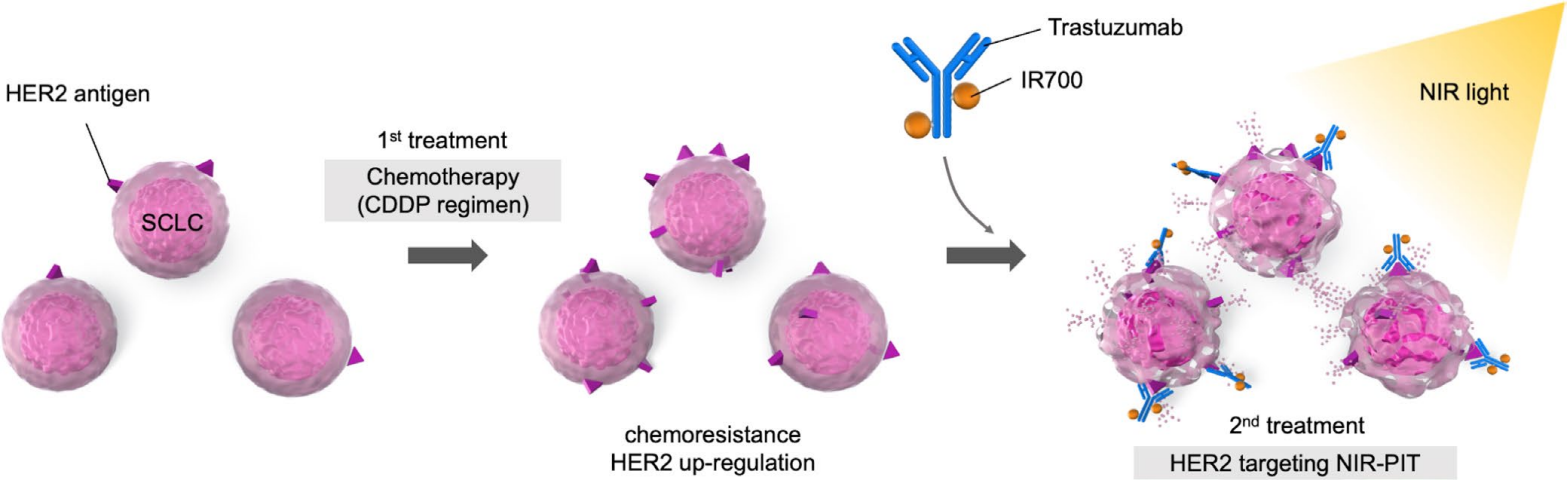
Scheme of HER2‐targeting NIR‐PIT for HER2‐positive CDDP‐resistant SCLC. When SCLC acquired chemoresistance to CDDP‐based therapy as the standard first line and HER2 expression is upregulated, HER2‐targeting NIR‐PIT using tra‐IR700 has a rational advantage for the second line or later. CDDP, cisplatin; HER2, human epidermal growth factor receptor 2; NIR‐PIT, near‐infrared photoimmunotherapy; SCLC, small cell lung cancer

We demonstrated the HER2‐targeting NIR‐PIT effect both in vitro and in vivo using tra‐IR700 for HER2‐positive SCLC. HER2 expression was higher in CDDP‐resistant SCLC cells than in parental cells, and the antitumor effect of HER2‐targeting NIR‐PIT was higher in CDDP‐resistant SCLC, suggesting the HER2 expression level was positively correlated with the efficacy of NIR‐PIT. Since CDDP‐based chemotherapy is the standard first line in SCLC,^5^, ^6^, ^7^ HER2‐targeting NIR‐PIT could be a choice following the failure of the CDDP regimen for HER2‐positive SCLC (Figure 5).

The targeting HER2 therapeutic agents that have already been used in the clinic are classified into three types. First, trastuzumab and pertuzumab are the monoclonal antibodies and they inhibit cell signals or dimer formations with another ErbB family receptors, respectively.^37^, ^38^, ^39^ Second, trastuzumab emtansine and trastuzumab deruxtecan are the ADCs that are synthesized by binding a payload to an antibody via a linker, and they release anticancer agents after being internalized and catabolized.^40^, ^41^ Finally, small‐molecule drugs, lapatinib, neratinib, and tucatinib, are tyrosine kinase inhibitors, which hinders the cell proliferative signals.^42^, ^43^, ^44^ Though these molecularly targeted drugs could be available for HER2, no targeted therapy has yet been approved for SCLC.^30^

According to this study, HER2‐targeting NIR‐PIT using trastuzumab might be a targeted therapy for HER2‐positive SCLC. The efficacy of HER2‐targeting NIR‐PIT has also been reported in NSCLC,^45^ gastric cancer,^46^ breast cancer,^47^ bladder cancer,^48^ and ovarian cancer^49^ in several preclinical research. Recent research revealed that the mechanism of NIR‐PIT was rapid necrotic cell death due to photochemical ligand reactions. This photochemical reaction changes the hydrophilic side chains (silanol) of IR700 into hydrophobic, which introduces the aggregation of the antibody‐IR700 conjugates. With this unique mechanism for specific cell membrane ruptures, NIR‐PIT is thought to be a new modality in cancer therapy.^50^ Thus, it is meaningful to develop this technology for intractable cancers with limited treatment options.

Although several reports had been reported that HER2 is upregulated in chemoresistant cells compared with SCLC parent cells, the details of the mechanism of HER2 have not been fully clarified. HER2 was upregulated in SCLC chemoresistant cells and that the resistance could be overcome by combination therapy with lapatinib, an EGFR/HER2 tyrosine kinase inhibitor, and cytotoxic agents.^12^ Downregulation of miR‐125b and miR‐125a by cytotoxic drugs could be the part of the mechanism in HER2 upregulation in SCLC and proposed a therapeutic strategy using trastuzumab in combination with cytotoxic drugs.^14^ We also found that HER2 was upregulated in SCLC chemoresistant cells, especially in CDDP‐resistant cells, and it could be an advantage for HER2‐targeting NIR‐PIT. Previous studies reported a correlation between antigen expression and the therapeutic effect of NIR‐PIT by comparing completely different types of cell lines with the different cytoskeleton, antigen expression, and properties.^51^ On the other hand, in our study, we used parental and its chemoresistance of the same cell line. The basic properties in both cells are thought to be very similar. Therefore, we can suggest more clearly than before that the difference in therapeutic efficacy of NIR‐PIT is related to the antigen level. Based on these results, we considered the following as an example of the treatment strategy for HER2‐positive SCLC. First, SCLC is usually sensitive to cytotoxic agents, and CDDP‐based chemotherapy should be administered as a first‐line treatment. If the patient becomes resistant to chemotherapy (usually resistant to CDDP), HER2‐targeting NIR‐PIT has a rational advantage in the second‐line or later treatment for HER2‐positive SCLC.

There are some concerns in this study. First, if we irradiate lung cancer from outside the body, the NIR light will not reach the lesion. We investigated the use of bronchoscopy or endobronchial ultrasonography to bring the light source closer to the lesion. Second, we expect the indirect antitumor effects of NIR‐PIT for lesions and metastases that are difficult to irradiate directly. NIR‐PIT induces rapid, cellular necrosis that activates dendritic cells and promotes the autoimmune cycle.^52^ In addition, it has been reported that downregulating intratumoral Tregs enhance immunogenic cell death by suppressing cancer immune escape, and that anti‐tumor effects were observed even in lesions that were not directly irradiated.^53^ These treatment strategies may be effective in SCLC which rapidly progress and metastasize. The recent development of HER2‐targeting ADCs (i.e., trastuzumab emtansine and trastuzumab deruxtecan) could be used as a strategy which the main tumor lesion was treated with NIR‐PIT, and small metastases are with ADC itself.

In conclusion, NIR‐PIT targeting HER2 with trastuzumab showed a sufficient antitumor effect and was feasible for HER2‐positive SCLC. HER2 expression is increased when HER2‐positive SCLC cells acquire chemoresistance, especially CDDP resistance, and the efficacy of NIR‐PIT was significantly higher when SCLC cells acquired CDDP resistance. These findings suggest a positive correlation between HER2 expression and the NIR‐PIT effect. NIR‐PIT‐targeting HER2 is expected to be a novel therapeutic approach after CDDP‐based chemotherapy for HER2‐positive SCLC.

## CONFLICT OF INTEREST

The authors declare that they have no conflict of interest.

## ETHICS STATEMENT

All procedures followed were in accordance with the ethical standards of the responsible committee on human experimentation (institutional and national) and with the Declaration of Helsinki 1964 and later versions.

## Supporting information

FIGURE S1

## Data Availability

The data sets used and analyzed during the current study are available from the corresponding author on reasonable request.
