# Supplementary figures and images for: HER2 targeting near‐infrared photoimmunotherapy for a CDDP‐resistant small‐cell lung cancer
Source: Cancer Med. 2021 Nov 2;10(24):8808–19. doi: 10.1002/cam4.4381 (PMC8683547; doi:10.1002/cam4.4381)

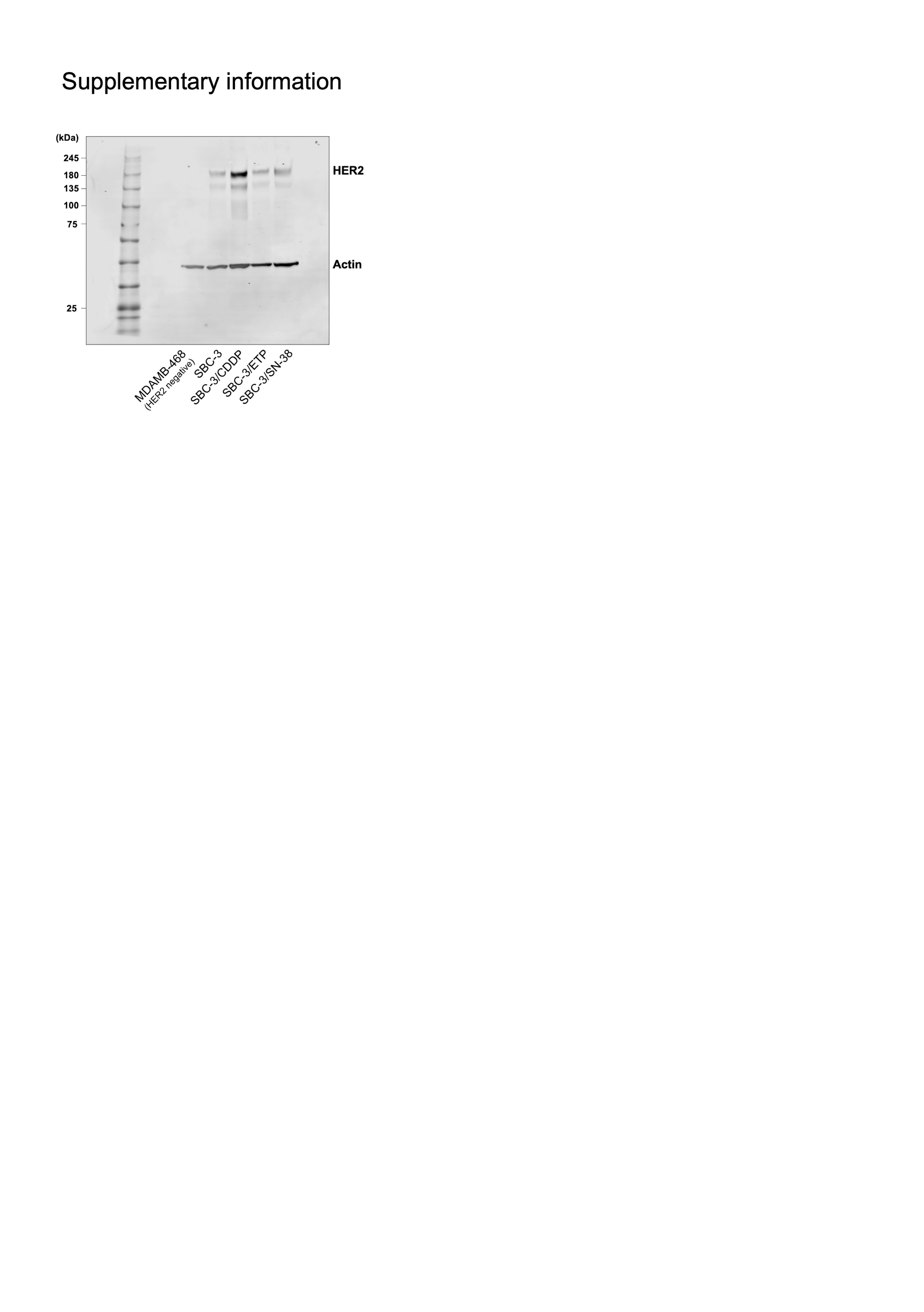

Supplement: Supplementary file 1 — FIGURE S1 [file CAM4-10-8808-s001.png]
